# They Are Not All the Same: Defenders of Ethnically Victimized Adolescents

**DOI:** 10.1007/s10964-024-02026-2

**Published:** 2024-06-06

**Authors:** Pinar Bilir Özturk, Sevgi Bayram Özdemir, Dagmar Strohmeier

**Affiliations:** 1 Center for Lifespan Development Research, School of Behavioural, Social and Legal Sciences, Örebro University, Örebro, Sweden; 2 School of Medical Engineering and Applied Social Sciences, University of Applied Sciences Upper Austria, Linz, Austria; 3 Norwegian Centre for Learning Environment and Behavioural Research in Education, University of Stavanger, Stavanger, Norway

**Keywords:** Ethnic victimization, Defending, Perspective taking skills, Diversity norms, Early adolescence

## Abstract

Developing a comprehensive understanding of adolescents’ defending behaviors in peer victimization incidents is crucial, as these behaviors are instrumental in preventing victimization in schools. Despite recent efforts to examine various defender subgroups and their characteristics, the heterogeneity in defending behaviors within the context of ethnic victimization remains unclear. To address this gap in knowledge, the current study examined naturally occurring subgroups of defenders in ethnic victimization incidents and investigated whether these subgroups differ in their socio-cognitive skills, class norms, and social status within peer relationships. The sample included adolescents in Sweden (*N* = 1065; *M*_*age*_ = 13.12, *SD* = 0.41; 44.5% females). Cluster analysis yielded four distinct subgroups: *victim-oriented defenders* (41.3%), *hybrid defenders* (23.5%), *bully-oriented defenders* (9.8%), and *non-defenders* (25.4%). Hybrid and victim-oriented defenders had higher levels of perspective taking skills and positive attitudes toward immigrants than non-defenders. All three defender subgroups perceived their classroom climate as more socially cohesive than non-defenders. All four subgroups did not significantly differ in their peer status. These findings emphasize the importance of fostering inclusive class norms and implementing classroom practices that facilitate the development of perspective taking skills among students. Such effort can enhance adolescents’ active defending behaviors in instances of ethnic victimization.

## Introduction

Along with the increasing migration rates all around the world, ethnic victimization has become a salient issue in schools. A recent report focusing on 5th grade students in Sweden showed that 48% of the adolescents reported witnessing racist harassment in their school at least once (Rädda Barnen, 2021). Sweden is a multi-ethnic country, where almost 21% of its citizens are born outside of Sweden. Among children and adolescents in compulsory education (aged 6 to 15), 11.36% are foreign-born students (SCB, 2024). Despite being recognized for its successful integration policies, such as citizenship access, family reunion, labor market mobility, and accessible healthcare and education (MIPEX, 2020), Sweden has also witnessed increased polarization, mirroring trends in other European countries, over the past decade. Far-right political parties with anti-immigrant discourses are gaining popularity and media narratives highlighting shortcomings in Swedish immigration policies have contributed to negative public perceptions of immigration and ethnic diversity (Munobwa et al., 2021). These factors are likely to influence social dynamics in schools, potentially contributing to incidents of ethnic victimization and shaping bystander behaviors in schools.

Adolescents vary from one another on how they respond to ethnic victimization incidents. Some adolescents may take actions to defend the targets whereas others may do nothing and stay passive (Malti et al., 2015). Defenders’ actions may give an implicit message to perpetrators about the (un)acceptability of their behaviors and have the possibility to influence the social dynamics in inter-ethnic interactions. Many of the studies in the peer victimization (e.g., Jenkins & Fredrick, 2017; Pozzoli et al., 2017) and ethnic victimization literature (e.g., Abbott & Cameron, 2014) consider defenders as relatively uniform groups. However, there is an increasing awareness that defenders are not a homogenous group (e.g., Reijntjes et al., 2016; Yun, 2020). For example, a Dutch study on fourth grade students identified three distinct subgroups of defending behaviors in reaction to bullying incidents: a subgroup who adopts strategies directed at perpetrators, a subgroup who adopts strategies directed at the targets and a subgroup who uses a combined strategy (Reijntjes et al., 2016). It is reasonable to assume that a similar heterogeneity may also exist among defenders of ethnic victimization, but literature on this topic is largely absent. In the present study, the aim was to identify different subgroups of defenders in ethnic victimization incidents. Additionally, the study investigated whether the identified defender subgroups differ regarding demographic (i.e., gender and immigrant status), individual (i.e., perspective taking skills and attitudes toward immigrants), contextual (i.e., inter-ethnic class norms), and interpersonal factors (i.e., peer acceptance).

### Heterogeneity in Defending Behaviors

Defending consists of several prosocial behaviors that are carried out with the intent to help victimized individuals (Lambe & Craig, 2020). Adolescents who defend their harassed peers have high levels of socio-cognitive and socio-emotional skills. Compared to passive onlookers, adolescents who include an excluded target in their group, do not only judge the perpetrators’ behavior as wrong because of fairness and empathetic reasons, but are also able to recognize the targets’ distressed emotional states (Malti et al., 2015). Defenders are also able to infer that the negative emotions of the targets are presumably caused by the harassments carried out by the perpetrators (Geraci & Franchin, 2021). However, defenders vary from one another regarding their concrete behaviors (Garandeau et al., 2022; Reijntjes et al., 2016). For example, in a study focusing on general victimization of ten year old children in the Netherlands (Reijntjes et al., 2016), three distinct subgroups of defenders were identified based on peer nomination data: *(a) victim-oriented defenders* who adopt altruistically motivated behaviors such as comforting victims and being friendly to them, *(b) bully-oriented defenders* who engage in goal-directed behaviors such as verbally or physically trying to stop the perpetrators, *(c) overall defenders* who concern for victims’ well-being and adopt goal-directed behaviors to put an end to the bullying. Additionally, another group identified is referred to as *(d) non-defenders* who do not engage in any type of defending behaviors. Non-defenders were found to constitute the largest group (52%), followed by victim-oriented (25%), bully-oriented (13%), and overall defenders (10%). Further, the majority of victim-oriented defenders and overall defenders consisted of girls whereas boys were the majority among bully-oriented defenders and non-defenders.

Only very few studies investigated the behaviors exhibited by witnesses of ethnic victimization. The available studies have focused either on one type of defending behavior at a time (e.g., Bayram Özdemir et al., 2022; Gönültaş & Mulvey, 2021) or conceptualized defending as an overarching category and overlooked the possible heterogeneity in defending behaviors (Abbott & Cameron, 2014). It is important to examine the heterogeneity in defending behaviors to facilitate the development of more effective prevention programs. Relying on the findings of previous research focusing on general peer victimization (Reijntjes et al., 2016), it was hypothesized that adolescents might also differ from one another regarding their responses to ethnic victimization incidents. It was expected that there would be at least three subgroups of defenders. There would be hybrid defenders who adopt strategies aiming to stop the perpetrator as well as to comfort the victim. There would also be a subgroup of adolescents who just confront the perpetrator and one third subgroup of adolescents who just comfort the victim. In addition to examining heterogeneity among defenders in ethnic victimization incidents, the present study also aimed to investigate the characteristics of different defender subgroups by focusing on various demographic (i.e., gender and immigrant status), individual (i.e., perspective taking skills and attitudes toward immigrants), interpersonal (i.e., peer acceptance) and contextual factors (i.e., inter-ethnic class norms).

### Perspective Taking and Defending Behaviors

Perspective taking represents the cognitive component of empathy and is responsible for the understanding of other persons’ perspectives, whereas empathic concern is the emotional component of empathy and captures the ability to feel other persons’ experiences (Eisenberg et al., 2010). Adolescents differ from one another regarding the extent to which they interpret social cues in peer interactions and how they understand and feel the emotions of victims. Their socio-cognitive and socio-emotional skill differences result in variations in their responses to victimization incidents at school (e.g., Pozzoli et al., 2017; Wolgast et al., 2022). For example, both high levels of perspective taking skills and empathic concern were found to be positively associated with defending behaviors (Gini et al., 2008; Pozzoli et al., 2017), whereas low levels of empathic concern and perspective taking skills were related to bullying behaviors among adolescents (Gini et al., 2008). However, only perspective taking skills, rather than empathic concern, were found to be associated with willingness to intervene in another study (Espelage et al., 2012).

Perspective taking and empathic concern also play an essential role in how young people with diverse background perceive and interact with each other (Bayram Özdemir et al., 2020) and how they respond to bias-based victimization incidents (Abbott & Cameron, 2014; Gönültaş & Mulvey, 2023). For example, adolescents with higher levels of empathic skills have a greater tendency to evaluate bias-based victimization as less acceptable (Gönültaş & Mulvey, 2023), and they are also more likely to challenge the perpetrator by taking assertive actions (Abbot & Cameron, 2014). Together, these findings suggest that higher levels of perspective taking skills might help young people to have a good understanding about the possible impact of ethnic victimization on targets, and this in turn may impact their actions. However, the literature remains unclear regarding the possible role of adolescents’ perspective taking skills across different defender groups. Namely, it is unknown whether adolescents who employ different defending behaviors differ in their perspective taking skills. Such knowledge is crucial for developing an advanced understanding regarding the possible socio-cognitive skills of adolescents in different defender subgroups. Based on the available literature, it was expected that defenders would be more likely to have higher levels of perspective taking skills compared to non-defenders. However, no specific hypothesis was proposed regarding how defender subgroups would differ from each other in terms of their perspective taking skills, partly due to lack of knowledge in the literature. Such examinations will be exploratory in nature.

### Attitudes Toward Immigrants and Defending Behaviors

The developmental intergroup approach highlights that intergroup processes (e.g., intergroup contact, group affiliation, in-group and out-group attitudes) shape how children and adolescents reason about and respond to social exclusion and conflicts in diverse settings and these processes are influenced by the development of social-cognitive abilities (Bigler & Liben, 2006; Palmer & Abbott, 2018). Using mostly experimental paradigms, empirical studies supported the premises of this theoretical framework (e.g., Gönültaş & Mulvey, 2021; Palmer et al., 2022). For example, a study examined whether minority status (immigrant versus non-immigrant) contributed to preadolescents’ bystander reactions, and reasoning about intergroup social exclusion in Cyprus (Palmer et al., 2022). It was found that non-immigrant preadolescents reported higher levels of prosocial bystander behaviors (e.g., reporting to the teacher, comforting the excluded target) when non-immigrant victims were excluded compared with when immigrant victims were excluded. Importantly, they also found that non-immigrant preadolescents with high levels of intergroup contact experiences reported higher levels of prosocial bystander behaviors for immigrant victims compared to those with lower levels of intergroup contact experiences. Similarly, another study examined how preadolescents and adolescents judged general and bias-based bullying and examined how likely they would intervene if they witnessed bullying of immigrant-origin and non-immigrant-origin peers in three hypothetical scenarios (Gönültaş & Mulvey, 2021). It was found that immigrant adolescents evaluated both general and bias-based bullying as more unacceptable when the victim was immigrant compared to their non-immigrant origin classmates. Further, non-immigrant adolescents (compared to immigrant adolescents) were more likely to report that they would intervene (i.e., say something to the aggressor; seek help) when the victim was a non-immigrant-origin adolescent.

Beyond social group affiliations, research using the developmental intergroup approach as a theoretical framework also showed that children and adolescents’ views about diversity (Abbott & Cameron, 2014), their desired social distance with outgroup members (Gönültaş & Mulvey, 2023), and their attitudes toward out-group members (e.g., Gönültaş & Mulvey, 2023; Hitti et al., 2023) contribute to their bystander behaviors in intergroup social exclusion and bias-based bullying. More specifically, adolescents had a greater intention to defend their victimized peers (e.g., by comforting the victim, asking the bully to stop) when they reported higher levels of desire for contact with immigrants (Hitti et al., 2023) and had positive intergroup contact experiences (Abbott & Cameron, 2014). On the other hand, adolescents with higher levels of desired social distance and prejudicial or discriminative attitudes toward out-group members were more likely to perceive ethnic victimization as acceptable. They also had a greater tendency to support the bully (Gönültaş & Mulvey, 2023), and were less likely to intervene (Hitti et al., 2023). In sum, the impact of inter-group processes (particularly social group membership and intergroup contact) on adolescent’s bystander behaviors has been well studied in the literature. However, there is a lack of empirical studies regarding how adolescents’ interethnic attitudes (which is an important component of intergroup processes and an essential indicator of intergroup relationships) are related to their engagement in different forms of defending behaviors. Developing such an understanding will shed light on the complex association between adolescents’ attitudes and their actions in inter-group relations, and in turn will provide a valuable information to school personnel in their anti-bullying efforts to prevent ethnic victimization e.g., by preparing teacher-initiated discussions regarding openness to diversity and cultural understanding to support students’ positive attitudes toward immigrants. Based on the available literature, it was expected that adolescents in any defender subgroup would have higher positive attitudes toward immigrants than non-defenders. In addition, differences in attitudes toward immigrants across defender subgroups will be explored.

### Inter-Ethnic Class Norms and Defending Behaviors

The social-ecological model of peer victimization (Swearer & Espelage, 2010) highlights that students’ responses to peer victimization incidents are also context dependent. Students often act in line with the norms and atmosphere of their school and classroom. Therefore, social norms existing in a class context can support or inhibit adolescents’ tendencies to behave in particular ways. Supporting these arguments, several studies have shown that approximately 10–35% of the variance in defending behaviors can be explained by the differences between classes (Pozzoli et al., 2012; Salmivalli et al., 2011). A social ecological review of peer defending (Lambe et al., 2019) concluded that high levels of anti-bullying class attitudes and norms are associated with high levels of defending. Specifically, studies on general victimization reported that bystanders chose to defend their victimized peers more by trying to stop bullying (Xie & Ngai, 2020) and comforting the victimized peers (Thornberg et al., 2022), if they perceived that their classmates highly endorse anti-bullying norms. Another study (Yun, 2020) found that anti-bullying norms in a class especially influence adolescents who are already sympathetic to victims of bullying for taking any action to comfort them. Moreover, if students perceived that intervening in bullying situations is the norm in class, they were more likely to become victim-oriented defenders. Another study (Salmivalli & Voeten, 2004) however showed there was no association between antibullying class norms and secondary students’ defending behaviors in bullying except for girls in grade six. Together, these findings highlight that class norms might play a crucial role on adolescents’ responses in a peer victimization incident.

In addition to established relations between class norms and defending behavior in general bullying, class and school norms also play a role in how early adolescents perceive their peers who are different from themselves (Bayram Özdemir et al., 2021), the way they interact with each other in diverse social settings (Bayram Özdemir & Özdemir, 2020), and how they respond to bias-based victimization incidents (Bayram Özdemir et al., 2022). For example, early adolescents were found to have a greater intention to comfort their ethnically victimized classmates in classes where students respect each other’s cultural values and where they cooperate with each other in different class activities (Bayram Özdemir et al., 2022). Because of these established associations of class norms and defending behavior, the study aimed to develop an even more nuanced understanding by exploring whether different defender subgroups perceive different levels of positive inter-ethnic norms in their class.

### Peer Acceptance and Defending Behaviors

It is widely accepted that peer relationships make a unique contribution to social and emotional development of adolescents (Brown & Larson, 2009), and contribute to their social capital (Putnam, 1995). Adolescents with more social ties often are likely to have access to information and to have social influence and credentials in their peer contexts. Such social advantages might facilitate their engagement in assertive and prosocial behaviors when they witness bullying (Garandeau et al., 2022). Supporting this conceptual argument, a study (Caravita et al., 2009) argued that adolescents with a high status (i.e., socially preferred or having high popularity and peer acceptance) might have a secure position in the class. Standing up to the bully might not put them at risk of being the next victim to the same extent as those with a low peer status. Thus, they might be more ready to adopt defending behaviors. And indeed, socially preferred children and adolescents and popular children in the class were the ones who often defended victims (Caravita et al., 2009). Studies focusing on different forms of defending behaviors; however, showed that associations between peer status and defending behaviors might vary depending on how peer status is operationalized. Specifically, a study (Reijntjes et al., 2016) reported that overall defenders and bully-oriented defenders were perceived more popular than victim-oriented defenders and non-defenders whereas victim-oriented defenders and overall defenders were more liked by the peers than bully-oriented defenders and non-defenders. It is possible that adolescents with high popularity might believe that they have the power of influencing others in their social network and their decisions and actions are supported by their peers. Thus, they might not foresee any social risk of adopting strategies directed at perpetrators. On the other hand, adolescents who are liked by their classmates may give importance to friendships and accordingly engage in defending behaviors to show that they care for the victim.

Social status in peer relationships seems to provide adolescents a shield against the possible risks that their defending decision might pose. However, the role of peer relationships (particularly peer acceptance) for defenders of ethnic victimization remains unclear in the literature. As in general peer victimization, adolescents’ peer status might also contribute to their actions in inter-ethnic relationships. It is possible that adolescents who are well accepted by their classmates might have a high status in social relationships, and thus have great courage for standing up to a bully. Relatedly, it was expected that bully-oriented defenders and hybrid defenders who employ high levels of bully-oriented defending strategies may have higher levels of peer acceptance compared to the other groups.

## Current Study

Developing a comprehensive understanding of adolescents’ defending behaviors in peer victimization incidents is crucial, as these behaviors are instrumental in preventing victimization in schools. Despite recent efforts to examine various defender subgroups and their characteristics, the heterogeneity in defending behaviors within negative inter-group relationships, particularly during early adolescence, remains unexplored. Hence, the current study aimed to develop a more nuanced understanding about early adolescents’ responses to ethnic victimization incidents at school in Sweden. Specifically, the first aim of the current study was, by using a person-centered approach, to investigate whether there are distinct groups of adolescents who engage in different forms of defending behaviors in ethnic victimization incidents. Based on previous research, it was expected that there would be four different groups: hybrid defenders, victim-oriented defenders, bully-oriented defenders, and non-defenders. The second goal was to examine whether the groups who exhibit different responses to ethnic-victimization incidences display different levels of perspective taking skills, positive attitudes toward immigrants, positive inter-ethnic class norms, and peer acceptance. Relying on the aforementioned conceptual arguments and empirical studies in the literature, it was hypothesized that defenders would have higher levels of perspective taking skills, positive attitudes toward immigrants, and positive inter-ethnic class norms compared to non-defenders. It was also expected that there might be differences among defender subgroups. However, the literature on this topic is widely absent, and thus this question was examined in an exploratory manner. Regarding peer acceptance, it was hypothesized that bully-oriented defenders and hybrid defenders would have a higher status in peer relationships compared to the other groups.

## Methods

### Participants

The sample of the present study comes from the first wave of a 3-year longitudinal study, the Youth and Diversity Project, which has been implemented in 55 classrooms in 16 different schools in central Sweden. The target sample of the current study consisted of 1286 seventh grade students. In Sweden, the curriculum for seventh graders includes a number of compulsory courses, along with some elective ones. Typically, students remain with the same classmates for the majority of these courses, except for a few optional courses where they may be divided into groups based on their interests and move to different classes. That is, students largely stay within the same classroom environment. About 17% of the target sample did not take part in the study due to various reasons, such as parental disapproval, lack of student consent, and student absence during data collection. The final sample consists of 1065 adolescents (*M*_*age*_ = 13.12, *SD* = 0.41; 44.5% females; 17.5% born outside of Sweden). More than half of the youth (59%) had parents who were born in Sweden (defined as Swedish adolescents), 13% of them had one parent who was born outside Sweden (defined as adolescents with mixed background), and 28% of them had both parents born outside Sweden (defined as adolescents with immigrant background). The parents of these adolescents had come to Sweden from around sixty diverse nations, including Syria, Turkey, Australia, Iraq, Thailand, the Netherlands, Colombia, Somalia, Morocco, Ukraine, Lebanon, Germany, Chechnya, and England. Both parents’ country of birth for classification was used because it enables to examine Swedish adolescents and adolescents with immigrant background but also those with mixed background. Classroom compositions varied across gender distribution (i.e., the proportion of girls ranged from 19% to 85%), parents’ employment status (i.e., the proportion of employed mothers ranged from 33% to 100%, and the proportion of employed fathers ranged from 62% to 100%) and proportion of students with at least with one parent born outside of Sweden (ranging from 12% to 100%). See Bayram-Özdemir and Özdemir, 2020 for more information about the characteristics of the sample.

### Procedure

The data collection process was overseen by a research manager and trained research assistants. Before the data collection, a letter was sent to parents in order to inform them about the project. During the data collection, students were first informed about the main aim of the study, the voluntary basis of their participation, and confidentiality of their answers. Thus, all students who were present at the day of data collection and who accepted to participate and whose parents did not decline their participation took part in the study. See Bayram-Özdemir and Özdemir, 2020 for more information about the study procedure.

### Measures

#### Adolescents’ intended responses to ethnic victimization

A stem question was provided to students (e.g., What would you do if one or more students at your school made fun of or teased another student because of her/his foreign appearance, ethnic background, or religion?”). Afterward, they were asked to rate their likelihood of intervening in the situation by responding to the following two statements formulated based on previous research (Salmivalli & Voeten, 2004): “I would try to comfort the student who is teased” and “I would tell the others to stop making fun of him or her.” Responses to these items were provided using a 5-point scale, ranging from “1” (not at all likely) to “5” (very likely). It is important to note that the causes of ethnic victimization are multifaceted, involving factors such as foreign appearance, cultural heritage, religion, speaking a different first language, and having an accent in European context (e.g., Strohmeier & Gradinger, 2023). Therefore, when formulating the stem question, a comprehensive approach was taken to encompass a wide array of potential reasons, rather than solely focusing on heritage background. Adolescents’ scores on the items were positively associated with their positive attitudes towards immigrants (r*s* ranged from .30 to .38) and negatively related to their engagement in ethnic victimization (r*s* ranged from −.18 to −.23), indicating the concurrent validity of the scale.

#### Perspective taking skills

The Interpersonal Reactivity Index was used to measure perspective-taking skills of adolescents in their peer relationships (Davis, 1980). The scale consists of five items (e.g., “I try to understand everyone’s point of view in a conflict before reaching a decision” and “I try to understand others better by imagining how things look from their perspective”). Adolescents were asked to report how well each statement described them on a 5-point Likert scale, ranging from “1” (doesn’t describe me well at all) to “5” (describes me very well). Internal consistency and test-retest reliability of the scale were found to be good (Davis, 1980). In the present study, Cronbach’s alpha for the scale was 0.79.

#### Adolescents’ positive attitudes toward immigrants

The Tolerance and Xenophobia Scale (van Zalk et al., 2013) was used to assess adolescents’ positive attitudes towards immigrants. This scale comprises six items (e.g., “Our culture benefits from the presence of immigrants” and “Immigrants should be allowed to maintain their own customs and lifestyle”). Participants were asked to indicate their level of agreement or disagreement with these statements using a 5-point Likert scale, ranging from “1” (strongly disagree) to “5” (strongly agree). The scale has demonstrated strong internal consistency and predictive validity (van Zalk et al., 2013). In this study, Cronbach’s alpha for the scale was 0.84.

#### Positive inter-ethnic contact norms and cooperation in class

A revised version of the Classroom Cultural Diversity Climate scale was used to assess the perceived positive inter-ethnic contact norms and cooperation within the class (Bayram Özdemir & Özdemir, 2020; Schachner et al., 2021). Adolescents were provided with a set of five statements (e.g., “Students in my class interact harmoniously despite our diverse ethnic/cultural backgrounds” and “Students with different ethnic backgrounds in my class are friends with each other”) and asked to evaluate the extent to which these statements are true within their classroom setting. Adolescents evaluated each statement on a 5-point scale, ranging from “1” (not true at all) to “5” (completely true). In this study, the Cronbach’s alpha coefficient for the scale was 0.81.

#### Peer acceptance

A peer nomination method was used to assess adolescents’ peer acceptance. Students were asked to nominate up to five classmates with whom they are together most of the time. Nominations received from all classmates were used to compute peer acceptance scores for each child. The scores were standardized within each class (which typically include 20 to 30 students) to adjust for the differences in the number of nominators, and 6% of participants did not get any nomination.

#### Classroom ethnic composition

The classroom’s ethnic composition was created by computing the percentage of students with immigrant background (i.e., having at least one parent who was born outside Sweden) in each classroom. The proportion varied from 12% to 100% across a total of 55 classrooms.

### Data Analysis

Hierarchical cluster analysis with squared Euclidian distance employing the Ward method (Bergman, 1998) was applied to examine whether there were different subgroups of defenders. This analytical method was chosen due to its flexibility regarding distributional assumptions and its effectiveness with skewed data (Yim & Ramdeen, 2015), a pertinent consideration given that the variables that were included in the cluster analysis had slightly skewed distribution in this study. Additionally, although four potential clusters were expected, there were no a priori expectations regarding the within-group variations. Hierarchical cluster analysis is not constrained by any specific within-group variations; rather it groups observations based on their observed similarities and distances (Yim & Ramdeen, 2015), which align with the aim of exploring naturally occurring subgroups among defenders.

Three statistical criteria were used to decide on the number of cluster solutions (Hair et al., 2018) in addition to conceptual considerations. First, large increases in the agglomeration coefficients were determined by using a scree plot. Second, the explained error sum of squares for each cluster solution was calculated (minimum suggested value of 67%, Bergman, 1998). Finally, homogeneity within clusters and heterogeneity across clusters were examined. Since cluster analysis is highly sensitive to extreme values, the z-transformed values of confronting the bully (3.1%) and comforting the victim (2.8%) that were less than −2.5 were recoded into the value −2.5 before estimating the cluster analysis (Bergman, 1998). Multivariate analysis of variance (MANOVA) was conducted to compare adolescents in different clusters on their levels of perspective taking skills, attitudes toward immigrants, peer acceptance, and inter-ethnic class norms.

### Missing Data

More than two-thirds of the sample (79.2%) had complete data in all study variables. Missingness across the study variables varied from 0.2% to 14.55%. Little’s MCAR test was conducted using all study variables, including demographics (i.e., age, gender, country of birth, parents’ country of birth, parents’ work status, perceived family income, and whether the participant came from an intact family or not). The test was statistically significant, *χ*^*2*^(198) = 314.63, *p* = 0.001, indicating that missingness was not completely at random. A series of independent samples t-test were estimated to further investigate potential mechanism of missingness. Specifically, a dichotomous variable was created, with adolescents having complete data coded as 0 and those with at least one missing datum coded as 1. These groups were compared across all study variables. The results of t-tests showed that adolescents with complete data had higher levels of peer acceptance, reported greater positive inter-ethnic contact norms, and were more likely to confront bullies compared to those with at least one missing data. Considering these findings, the cluster analysis was conducted using both the dataset without missing data imputation and the dataset with missing data imputation (i.e., Expectation Maximization (EM) method). The results were similar. Therefore, in order to eliminate possible Type-II errors, the data with EM imputation was reported (Acock, 2005; Kline, 2011).

## Results

### Descriptive Analyses and Identification of Defender Subgroups

**Fig. 1:**
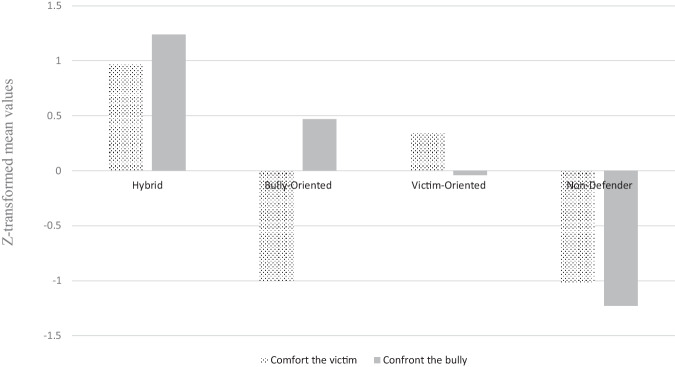
Four Defender Subgroups Based on the Mean Values of Defending Strategies

Means, standard deviations, and correlations among the study variables are presented in Table 1. Hierarchical cluster analysis was conducted to examine whether there were different groups of adolescents who engage in different forms of defending behaviors (i.e., confronting the bully and comforting the victim) in ethnic victimization incidents. A four-cluster solution was indicated by the point-of-inflection method (Hair et al., 2018). The four clusters explained 74% of the error sum of squares, which is higher than the minimum suggested value of 67% for identifying homogeneous clusters (Bergman, 1998). The homogeneity of variance assumption was violated. Hence, Welch *F*-tests were employed to examine cluster heterogeneity. The clusters were sufficiently differentiated on both confronting the bully, *F*(3, 361.22) = 642.96, *p* < 0.001, *η*^2^ = 0.68, and comforting the victim, *F*(3, 330.24) = 2516.49, *p* < 0.001, *η*^2^ = 0.79 (see Table 2 and Fig. 1). The differences between the clusters were examined using Sidak post-hoc comparison. The results showed the adolescents in the largest group, “victim-oriented defenders” (*n* = 440; 41.3% of the sample), reported high levels of comforting the victim and average levels of confronting the bully. Adolescents in the second largest group which was labeled as “non-defenders” (*n* = 271; 25.4% of the sample), reported low levels of confronting the bully and comforting the victim. The third largest group consisted of “hybrid defenders” (*n* = 250; 23.5% of the sample). Adolescents in this cluster reported high levels of engagement in both types of defending behaviors. The smallest group consisted of “bully-oriented defenders” (*n* = 104; 9.8% of the sample). Adolescents in this group displayed low levels of comforting the victim and high levels of confronting the bully.

**Table 1:** Bivariate correlations, means, and standard deviations of the study variables

	1	2	3	4	5	6	7	8	9
1. Gender^a^	–	0.002	−0.014	0.243***	0.143***	0.137***	0.095**	0.052	−0.052
2. Adolescents with mixed background^b^		–	−0.238***	0.031	−0.009	0.029	−0.003	0.027	−0.019
3. Adolescents with immigrant background^b^			–	0.071*	0.126***	0.017	0.104***	−0.067*	−0.183***
4. Comforting the victim				–	0.608***	0.398***	0.367***	0.258***	0.061*
5. Confronting the bully					–	0.317***	0.300***	0.221***	0.026
6. Perspective taking skills						–	0.371***	0.272***	0.053
7. Attitudes toward immigrants							–	0.326***	0.076*
8. Inter-ethnic class norms								–	0.107***
9. Peer acceptance									–
*M*				3.74	3.73	3.21	3.57	3.87	15.56
SD				0.93	1.02	0.70	0.76	0.65	9.19
Skewness				−0.68	−0.59	0.68	−0.63	−0.63	0.47
Kurtosis				0.56	−0.03	0.65	0.86	1.46	0.08
Min				1	1	1	1	1	0
Max				5	5	5	5	5	52.38

*p < 0.05, **p < 0.01, ***p < 0.001

^a^Gender was coded as 0 = Males and 1 = Females

^b^“Swedish adolescents” was defined as reference category

**Table 2:** Means, standard deviations, 95th percentile confidence intervals for comforting the victim and confronting the bully by cluster

			Comforting the victim			Confronting the bully		
	*N*	%	M	SD	95% CI	M	SD	95% CI
Cluster 1 - Hybrid	250	23.47	0.97^a^	0.51	0.91, 1.04	1.24^a^	0.70	1.23, 1.25
Cluster 2 - Bully-oriented	104	9.77	−1.01^c^	0.52	−1.11, −0.91	0.47^b^	0.40	0.39, 0.55
Cluster 3 - Victim-oriented	440	41.31	0.34^b^	0.42	0.30, 0.38	−0.04^c^	0.43	−0.08, −0.00
Cluster 4 - Non-defender	271	25.45	−1.02^c^	0.73	−1.12, −0.93	−1.23^d^	0.65	−1.31, −1.15
Welch *F*			642.96			2516.49		
*df*			361.335			330.241		
*p*			<0.001			<0.001		
*η*^2^			0.683			0.792		

^a, b, c, d^Superscripts denote the statistically significant group differences

### Gender and Immigrant Status Differences between Defender Subgroups

First, the associations between defender subgroups and adolescents’ gender and immigrant status were examined using chi-square tests (see Table 3). Girls were significantly more likely to be hybrid defenders and less likely to be bully-oriented defenders and non-defenders compared to boys. No gender differences were found for victim-oriented defenders. Swedish adolescents were significantly more likely to be non-defenders and less likely to be hybrid defenders. Adolescents with immigrant background, on the other hand, were significantly more likely to be hybrid defenders and less likely to be non-defenders. Moreover, adolescents with mixed background were significantly less likely to be bully-oriented defenders. Then, multilevel multinomial logistic regression analysis was estimated to examine whether the probability of being in one of the defending subgroups was related to classroom ethnic composition (i.e., proportion of students with at least one parent born outside of Sweden). Non-defender subgroup was defined as the reference category. The result showed that the probability of being in hybrid defender subgroup was higher in classroom with greater ethnic diversity compared to the probability of being in non-defender group, *b* = 1.14, *SE* = 0.52, *t* = 2.20, *p* = 0.03, OR = 3.13.

**Table 3:** Gender and Immigrant Status Differences between Defender Subgroups

	Gender		Immigrant status		
	Female	Male	Swedish adolescents	Adolescents with mixed background	Adolescents with immigrant background
Hybrid	142 (58%)	103 (42%)	126 (56.3%)	30 (12.2%)	89 (36.3%)
Bully-oriented	34 (32.7%)	70 (67.3%)	69 (66.3%)	6 (5.8%)	29 (27.9%)
Victim-oriented	221 (49%)	220 (51%)	241 (56.3%)	62 (14.5%)	125 (29.2%)
Non-defender	78 (29.3%)	188 (70.7%)	184 (62.2%)	36 (13.5%)	46 (17.3%)
*Chi-square*	52.123		30.87		
*df*	3		6		
*p*	<0.001		<0.001		

### Characteristics of Defender Subgroups

A 2 (gender) x 3 (immigrant status) x 4 (defender subgroups) MANOVA was estimated to test the multivariate effects of gender, immigrant status, and defender subgroups on the study variables, as well as the possible interaction effects. None of the interaction effects between gender, immigrant status, and defender subgroups were statistically significant: gender by immigration status interaction, *Pillai’s Trace* = 0.01, *F*(8, 2028) = 1.08, *p* = 0.38; gender by defender subgroups interaction, *Pillai’s Trace* = 0.01, *F*(12, 3045) = 0.48, *p* = 0.93; immigration status by defender subgroups interaction, *Pillai’s Trace* = 0.04, *F*(24, 4064) = 1.48, *p* = 0.06; gender by immigration status by defender subgroups, *Pillai’s Trace* = 0.02, *F*(24, 4064) = 0.73, *p* = 0.83. To reduce the complexity of MANOVA, the model was re-estimated by including only the main effects. There was a significant multivariate effect of defender subgroups, *Pillai’s Trace* = 0.15, *F*(12, 3096) = 13.99, *p* < 0.001, *η*^2^ = 0.05. Univariate analyses indicated that the defender groups significantly differed from each other on their levels of perspective taking skills and attitudes towards immigrants (see Table 4). Post-hoc tests revealed that hybrid defenders had higher levels of perspective taking skills and attitudes towards immigrants compared to all other groups. Bully-oriented defenders and non-defenders did not significantly differ from each other, but both groups scored lower than hybrid and victim-oriented defenders regarding perspective taking skills and attitudes towards immigrants.

**Table 4:** Characteristics of Defender Subgroups

	Hybrid		Bully-Oriented		Victim-Oriented		Non-Defender					
	M	SD	M	SD	M	SD	M	SD	*F*	*df*_*b*_*,df*_*w*_	*p*	η2
Perspective taking skills	3.50^a^	0.82	3.03^c^	0.71	3.31^b^	0.55	2.85^c^	0.60	42.648	3, 1033	<0.001	0.110
Attitudes toward immigrants	3.85^a^	0.77	3.43^c^	0.73	3.68^b^	0.62	3.22^c^	0.81	31.869	3, 1033	<0.001	0.085
Inter-ethnic class norms	4.04^a^	0.70	3.87^ab^	0.55	3.90^b^	0.61	3.69^c^	0.63	13.740	3, 1033	<0.001	0.038
Peer acceptance	16.26^a^	8.61	15.61^a^	8.92	15.06^a^	9.50	15.67^a^	9.24	1.971	3, 1033	0.117	0.006
*Box’s M*	391.439		*Pillai’s Trace*		0.154							
*F*	1.624			*F*	13.992							
*df*	220, 18769.786			*df*	12, 3096							
*p*	<0.001			*p*	<0.001							
				*η*^2^	0.051							

^a, b, c^Superscripts denote the statistically significant group differences based on Sidak

Regarding positive inter-ethnic contact norms and cooperation in class a slightly different pattern emerged. Non-defenders had the lowest levels of positive inter-ethnic contact norms compared with all other groups, while hybrid defenders had the highest levels. Interestingly, hybrid defenders did not differ from bully-oriented defenders who in turn did not differ from the victim-oriented defenders. Unlike the expectation, the four subgroups did not significantly differ from each other regarding their peer acceptance.

Further, there were significant multivariate effects for gender (*Pillai’s Trace* = 0.01, *F*(4, 1030) = 2.45, *p* = 0.04, *η*^2^ = 0.01) and for immigrant status (*Pillai’s Trace* = 0.06, *F*(8, 2062) = 7.87, *p* < 0.001, *η*^2^ = 0.03). Follow-up analyses showed that girls had higher levels of perspective taking skills and lower levels of peer acceptance compared to boys. Further, adolescents with immigrant background had lower levels of positive inter-ethnic contact norms and cooperation in class than Swedish adolescents, and their peer acceptance scores were significantly lower than both Swedish adolescents and adolescents with mixed background.

## Discussion

To date, defenders of ethnically victimized adolescents have been studied as a uniform group (Abbott & Cameron, 2014). However, distinct subgroups of defenders have been found for general peer victimization (Reijntjes et al., 2016; Yun, 2020). Thus, the present study aimed to extend the existing literature by examining whether there are also different defender subgroups in ethnic victimization incidents and whether these defender subgroups show variations in their socio-cognitive skills, views about immigrants, how they perceive their class context and social acceptance in peer relationships.

### Identification of Four Defender Subgroups

The first main discovery of the present study was that not all adolescents respond to ethnic victimization incidents in the same way. Instead, and in line with the literature on general victimization (Reijntjes et al., 2016), four defender subgroups were identified: victim-oriented defenders (41.3%), non-defenders (25.4%), hybrid defenders (23.5%) and bully-oriented defenders (9.8%). While victim-oriented defenders show high levels of comforting the victim, but low levels of confronting the bully, non-defenders show low levels on both variables. Hybrid defenders show high levels on both variables and bully-oriented defenders show high levels of confronting the bully, but low levels of comforting the victim. Compared with other studies focusing on general victimization (Reijntjes et al., 2016; Yun, 2020), the non-defender group was smaller in the present study, but in line with these previous studies victim-oriented defenders were a larger subgroup compared with hybrid (Reijntjes et al., 2016) and bully-oriented defenders (Reijntjes et al., 2016; Yun, 2020).

It is possible the smaller number of non-defenders that were found in the present study stem from different approaches of measurement. In the present study, adolescents were asked about their most likely reactions after presenting them a short incident covering foreign appearance, ethnic background and religion, as possible reasons for ethnic victimization. Conversely, in previous studies, youth were asked to evaluate their peers’ actual defending behaviors (Reijntjes et al., 2016) or to report their own defending behaviors (Yun, 2020). Thus, it is possible that the measurement approach of the present study produced some kind of desirability bias. Another explanation might be related to societal influences on moral development. It is possible that early adolescents in Sweden may evaluate ethnicity-based victimization as more unfair, morally wrong, or devastating than non-ethnicity-based victimization compared to early adolescents residing in other countries because of a strong emphasis on equality and the high-level endorsement of anti-discrimination policies in Sweden compared to other countries (MIPEX, 2020). If this holds true, early adolescents residing in Sweden might be more motivated to defend targets of ethnic victimization compared with early adolescents residing in other countries which in turn might explain why the non-defender subgroup was smaller in the present study. Future research could illuminate this possibility.

In line with previous research (Reijntjes et al., 2016; Yun, 2020), it was also found that more early adolescents are victim-oriented defenders compared to hybrid defenders or bully-oriented defenders. One possible explanation could be that when adolescents make a decision about supporting their victimized peers, they might perceive direct confrontation with the bully as riskier. Consequently, the majority of them might adopt strategies that involve defending the victim indirectly. This way, they convey an implicit message to the bully regarding the unacceptability of the behavior without directly challenging the bully. While a classical study of naturalistic observations of peer interventions on school playgrounds (Hawkins et al., 2001) found that defenders adopting victim-oriented strategies were as effective as those adopting bully-oriented interventions in stopping general peer victimization, future research should explore which subgroup of defenders are more likely to be effective in stopping ethnic victimization incidents at schools.

Importantly, gender differences were also found, indicating that girls are more often hybrid defenders whereas boys are more likely to be bully-oriented defenders and non-defenders. No gender differences were found for victim-oriented defenders. It is possible that the higher levels of perspective taking among girls might explain their higher involvement in hybrid defending and lower involvement in non-defending. Immigrant status also mattered for defender group membership: Swedish adolescents were more likely to be non-defenders and less likely to be hybrid defenders. Adolescents with immigrant background, on the other hand, were more likely to be hybrid defenders and less likely to be non-defenders. Lastly, adolescents with mixed background were significantly less likely to be bully-oriented defenders. Adolescents with immigrant background and mixed background undergo similar migration experiences. It is possible that they develop an understanding and empathy for each other’s encounters in the host society. This augmented awareness may help them become more responsive to negative events and therefore motivate them to adopt different strategies to defend their peers who are facing ethnic victimization.

### Characteristics of Defender Subgroups

The second main discovery of the present study is the identification of meaningful differences between the four defender subgroups regarding their socio-cognitive skills, perceptions about immigrants, and how they perceive their class context. Supporting the expectation and previous research on general bullying (e.g., Espelage et al., 2012; Pozzoli et al., 2017) and ethnic bullying (e.g., Abbot & Cameron, 2014; Gönültaş & Mulvey, 2023), it was found that hybrid and victim-oriented subgroups have higher levels of perspective taking skills compared to non-defenders. Interestingly, no difference was observed between bully-oriented defenders and non-defenders. As highlighted by the theory of prosocial moral behavior and development (Hoffman, 2001), perspective taking skills and empathy may enable young people to easily recognize the stress of victims. Assumed distress from victims may evoke a desire to alleviate the distress, thereby fostering adolescents’ involvement in defending behaviors. This could be one of the possible explanations of why hybrid and victim-oriented defenders have a higher level of perspective taking skills than bully-oriented defenders and non-defenders. Further, instead of focusing on the victim’s point of view, bully-oriented defenders may evaluate ethnic victimization through the lenses of fairness and social justice in their moral judgments. Thus, they may believe confronting the perpetrator directly is the most effective means to put an end to victimization and are unafraid of displaying their reactions.

Supporting the premises of the developmental intergroup approach (Bigler & Liben, 2006) and empirical studies (e.g., Bayram Özdemir et al., 2022; Gönültaş & Mulvey, 2023), the findings showed that adolescents’ defending behaviors are related to their attitudes toward immigrants. Specifically, it was found that hybrid defenders have the highest positive attitudes toward immigrants and are followed by the victim-oriented defender group. As expected, non-defenders had the lowest level of positive attitudes toward immigrants but did not significantly differ from bully-oriented defenders. It is likely that hybrid and victim-oriented defenders might perceive ethnic and cultural diversity as an opportunity to gain new perspectives rather than as a threat. Relatedly, they may have sufficient internal motivation to place themselves in a potentially risky position. On the other hand, non-defenders may find comfort in the belief that the world is just (i.e., Dalbert, 2009). They may therefore think that victims deserve to be treated in this way. Such feelings and viewpoints may impede these adolescents’ moral judgments about problematic inter-group relationships, leading them to choose not to intervene with the intention of defending victims.

Another important conclusion to draw from the findings is that adolescents’ perceptions of the class climate seem to be related with how they respond to ethnic victimization incidents. Supporting the arguments of social-ecological theory of peer victimization (Swearer & Espelage, 2010) and expanding previous research (e.g., Bayram Özdemir et al., 2022), it was found that all three defender subgroups were more likely to perceive their classroom climate as cooperative and socially cohesive compared to non-defenders. Further, hybrid defenders have a more positive perception about inter-ethnic norms in their class than victim-oriented defenders, but no difference was observed across hybrid and bully-oriented defenders. This finding underscores the significance of the classroom’s social climate in adopting more direct strategies (such as asking the bully to stop) to intervene in ethnic victimization incidents. It is possible that when youth are surrounded by peers who value diversity and embrace differences, they might find the courage to confront the bully because this supportive environment could decrease the potential costs associated with defending. Alternately, it is also plausible that inclusive and diversity-promoting class norms may foster the development of social-conventional reasoning in moral judgment among adolescents (i.e., ‘My group thinks it is not OK to bully someone because of her/his ethnic background’; Palmer et al., 2015). Through such reasoning, adolescents may perceive the cost of defending as low and consequently may choose to take an action. This finding, in fact, align with the broader bullying literature, suggesting that youth are more inclined to take on the role of defender when they are in a class context that devalues bullying (Lucas-Molina et al., 2018), emphasizes anti-bullying norms (Thornberg et al., 2022), and associates bullying with social costs (Peets et al., 2015). Alongside broader class norms, peer norms might play an important role in shaping adolescents’ values and defending behaviors during this developmental period. Despite some null findings (Gönültaş & Mulvey, 2021), research demonstrated that adolescents’ perceptions of their peers’ attitudes toward immigrants associated with a higher willingness to include victimized minority peers in their peer groups (Gönültaş & Mulvey, 2023) and a greater likelihood of standing against bias-based bullying (Hitti et al., 2023). Future studies should examine norms within adolescents’ close social networks to gain a deeper understanding of how norms regarding diversity in social relationships might influence adolescents’ defending behaviors.

Contrary to the expectation and previous research (Caravita et al., 2009), adolescents’ social status in peer contexts was not associated with their defending behaviors. All the four groups have similar levels of acceptance scores. Two alternative explanations can be provided for this finding. First, the role of overall peer acceptance across the four groups of defenders was investigated rather than differentiating peer acceptance by same versus cross ethnic peers. In fact, literature showed that bystanders’ judgments and responses in ethnic victimization differ based on whether the victim is an in- or outgroup member (Palmer et al., 2022). Given that overall peer acceptance seems not to play an important role on adolescents’ defending strategies, future studies should examine this relation by differentiating peer acceptance into intra- and interethnic acceptance. Another possible explanation could be that while defenders put themselves in a risky position by defending their victimized peers, they might not solely rely on the size of their social network. The characteristics of adolescents’ peer context (e.g., peers’ attitudes toward immigrants; van Zalk et al., 2013) and adolescents’ perceptions about the quality of their peer relations might contribute to their defending behaviors to a greater extent than their social status per se. It is possible that when early adolescents believe that they are surrounded by trusted peers, they may have the courage to challenge the bully because they may feel secure that they will be defended if they become the next target. Supporting this argument, literature showed that early adolescents (Jenkins & Nickerson, 2017) and adolescents (Evans & Smokowski, 2015) who perceived high level of support from their classmates were more likely to engage in prosocial bystander behavior in bullying situations. Therefore, these unmeasured factors might provide an explanation about the role of having secure and supportive social network in different defender groups. In sum, future research is needed to empirically examine these conceptual arguments, and to have a broader understanding about the role of peer relations on adolescents’ defending behaviors in ethnic victimization incidents.

### Study Limitations and Future Research

Given some noteworthy limitations of the present study, there are plenty of avenues for future research. First, adolescents’ intended defending behaviors were examined by using a hypothetical scenario about ethnic victimization. Although a similar method has already been used in previous studies (Gönültaş & Mulvey, 2021), it is well known that self-reported intentions are only modestly associated with actual reactions (Ajzen, 2005). Hence, future studies could examine to what extent the present findings hold true in real life ethnic victimization events. Second, the stem question used to measure adolescents’ defending behaviors combined various forms of victimization, such as foreign appearance, ethnic background, and religion, to comprehensively explore their responses to victimization stemming from different facets of their peers’ immigrant backgrounds. Although this approach has some value as the perceived reasons for ethnic victimization among defenders may vary depending on the situation, this approach may lack the specificity needed to address each form of victimization separately. Therefore, future research could consider examining different forms of ethnic victimization separately. Third, adolescents were grouped based on their parents’ country origin (i.e., born in versus outside of Sweden): Swedish adolescents, adolescents with mixed background, and adolescents with immigrant background. However, this approach may lack specificity as it treats adolescents with immigrant and mixed backgrounds as relatively homogeneous groups. Examining heterogeneity among adolescents based on factors such as their country origin, the reasons for migration, and their length of stay in the host country would provide a more nuanced understanding of defending behaviors and subgroup differences among adolescents originating from diverse backgrounds. Although large samples are needed to be able to differentiate these many possible sub-groups, such a differentiation could be an important contribution to the literature, especially considering that minority adolescents (i.e., Pakistani) were found to be victimized not only by their native peers but also certain ethnic groups (i.e., Hindu) (Eslea & Mukhtar, 2000). Thus, it is reasonable that adolescents belonging to certain religious or ethnic background groups might defend each other more. Fourth, two single items were used to measure each intended defending behavior. Such a measurement approach might limit the ability to capture nuances as well as variability in defending behaviors. Measuring different aspects of defending with multiple items is therefore suggested in future studies for a more thorough examination of defending behaviors among adolescents. Fifth, adolescents defined as non-defenders may consist of further subgroups, such as those who show high levels of reinforcing behaviors or those who show high levels of passive bystander behaviors. Future research is needed to examine possible subgroups within the non-defender group. Sixth, the longitudinal stability of the identified defender groups remains unknown. Longitudinal studies following adolescents over several years could illuminate these patterns. Seventh, the questionnaire did not disclose the gender of the bully when measuring adolescents’ defending behaviors. Future studies could consider specifying the gender of the bully to investigate its potential influence on respondents’ perceptions and responses. Last but not least, future studies could consider teachers’ inter-ethnic norms, because they have been found to be associated with defending behaviors (Priest et al., 2021).

## Conclusion

Much of the research examining defending behaviors in the context of ethnic victimization tends to consider defenders as relatively uniform groups. However, this approach overlooks potential subgroups among defenders as well as their unique characteristics. This study aims to address this gap in knowledge by examining naturally occurring subgroups among defenders of ethnic victimization incidents among adolescents. The results suggest that not all adolescents respond to ethnic victimization incidents in the same way, and subgroups show variations in various personal and contextual factors. Hybrid and victim-oriented defenders reported higher levels of perspective-taking abilities and favorable attitudes toward immigrants compared to non-defenders, who, on the other hand do not differ from bully-oriented defenders. Moreover, all three defender subgroups, particularly hybrid defenders, perceived their classroom environment as open to diverse vies diverse views, respecting each other’s cultural values, and cooperative, in comparison to non-defenders whereas they do not differ from the non-defender regarding their social status in peer relationship. Together, these findings highlight that instead of assuming all defenders are alike, these more refined distinctions among defenders can contribute to gaining a deeper understanding of the complex roles that defenders play in real ethnic victimization incidents within school settings. Further, the findings emphasize the importance of fostering inclusive class norms and implementing classroom practices that facilitate the development of perspective taking skills among students. Such an approach may contribute to adolescents’ nuanced understanding of the experiences of classmates from diverse cultures, and potentially motivate them to defend their victimized peers adopting both direct and indirect strategies.
